# Molecular Signatures of Nicotinoid-Pathogen Synergy in the Termite Gut

**DOI:** 10.1371/journal.pone.0123391

**Published:** 2015-04-02

**Authors:** Ruchira Sen, Rhitoban Raychoudhury, Yunpeng Cai, Yijun Sun, Verena-Ulrike Lietze, Brittany F. Peterson, Michael E. Scharf, Drion G. Boucias

**Affiliations:** 1 Dept. of Entomology, Purdue University, West Lafayette, IN, United States of America; 2 Interdisciplinary Center for Biotechnology Research, University of Florida, Gainesville, FL, United States of America; 3 Entomology and Nematology Department, University of Florida, Gainesville, FL, United States of America; Kansas State University, UNITED STATES

## Abstract

Previous studies in lower termites revealed unexpected synergies between nicotinoid insecticides and fungal entomopathogens. The present study investigated molecular mechanisms of nicotinoid-pathogen synergy in the lower termite *Reticulitermes flavipes*, using the nicotinoid, imidacloprid, in combination with fungal and bacterial entomopathogens. Particular focus was placed on metatranscriptome composition and microbial dynamics in the symbiont-rich termite gut, which houses diverse mixes of protists and bacteria. cDNA microarrays containing a mix of host and protist symbiont oligonucleotides were used to simultaneously assess termite and protist gene expression. Five treatments were compared that included single challenges with sublethal doses of fungi (*Metharizium anisopliae*), bacteria (*Serratia marcescens*) or imidacloprid, and dual challenges with fungi + imidacloprid or bacteria + imidacloprid. Our findings point towards protist dysbiosis and compromised social behavior, rather than suppression of stereotypical immune defense mechanisms, as the dominant factors underlying nicotinoid-pathogen synergy in termites. Also, greater impacts observed for the fungal pathogen than for the bacterial pathogen suggest that the rich bacterial symbiont community in the *R*. *flavipes* gut (>5000 species-level phylotypes) exists in an ecological balance that effectively excludes exogenous bacterial pathogens. These findings significantly advance our understanding of antimicrobial defenses in this important eusocial insect group, as well as provide novel insights into how nicotinoids can exert deleterious effects on social insect colonies.

## Introduction

Subterranean termites have lifestyles that are ideal for disease development. They live in moist, protected environments that are well suited for microbial growth, and because they are eusocial, colony members are in constant close contact. Despite these favorable conditions, termite epizootics are uncommon; to date, very few entomopathogens in nature have been discovered which infect these insects. The observed disease resistance is, in part, due to social behaviors that facilitate pathogen removal and transfer of resistance factors among nestmates [1,2]. For example, disruption of these behaviors by sublethal doses of neuro-pharmacological agents leads to dramatic increases in termite susceptibility to entomopathogens [3,4].

As do solitary insects, termites respond at the individual level to microbial pathogens by eliciting innate defense responses involving both cellular and humoral reactions [2]. Exposure of termites to sublethal pathogen challenges has been shown to trigger a defense reaction that produces sustained resistance to subsequent pathogen exposure [5,6]. Bulmer & Crozier [9] reported the presence of various pathogen- recognition proteins (PRPs) and the transcription factor *relish* in various termite species. Both *relish* and PRPs appear to be undergoing positive selection, suggesting a molecular arms race between pathogens and termite innate immune systems. In addition to the inducible innate response, certain termite species constitutively express antimicrobial peptides (AMPs) that display potent antifungal activity [7–9]. Analysis of gut transcriptome databases further suggests that termites have a functional innate immune response complete with a complex of recognition components, transcription factors, and AMPs [10,11]. However, components of the innate defense system may have multifunctional roles. For example, lysozyme, a known AMP and digestive enzyme, also can serve as an egg recognition pheromone in termite colonies [12], and gram-negative bacteria-binding Proteins (GNBPs) are structurally homologous to cellulases used by termites and other organisms for digesting their principal dietary component lignocellulose [13]. Additionally, endogenous endoglucanases well known for cellulose depolymerization have been shown to be inducible by pathogen challenge [11].

Another characteristic of termites is the presence of commensalistic microbiota in their digestive tracts that assist in lignocellulose digestion, nitrogen fixation, and intermediary metabolism [14]. How these commensals survive, multiply, and cycle through the termite gut via trophallaxis without triggering an antimicrobial response in the alimentary tract remains unclear. The lower termites, in particular, host diverse gut microbial communities consisting of both eukaryotes (protists) and prokaryotes (bacteria and archaea). Recent analyses of lower termites indicate that they can contain more than 12 protist species and more than 5,000 species-level bacterial phylotypes [15,16]. Moreover, because they are structural pests, lower termites are the intentional targets of many soil insecticides. One important group of soil termiticides is the nicotinoid class [17]. While effective for pest management and ectoparasite control, nicotinoids can have deleterious impacts on non-target species, in particular, honey bees [18,19].

**Fig 1:**
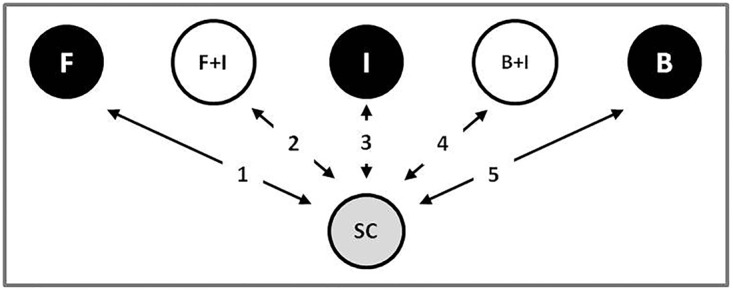
Microarray treatment summary: F (fungi), I (imidacloprid), B (bacteria), F+I (fungus + imidacloprid), B+I (bacteria + imidacloprid), and SC (solvent control) treatments. Reported analyses consisted of five pairwise comparisons to a common control, as denoted by the numbers 1–5.

Previously, the nicotinoid insecticide imidacloprid was found to greatly synergize the potency of fungal entomopathogens in the lower termite *Reticulitermes flavipes* [3]. Three hypotheses have been proposed as underlying causes of this synergy; namely, that imidacloprid suppresses: (a) social behaviors relating to grooming and trophallaxis, (b) gut symbiont populations, and/or (c) innate immune responses. Therefore, our goal here was to explore these hypotheses using an integrative approach combining imidacloprid and pathogen challenges at the whole-organism level with microarray analyses of gut metatranscriptome composition (Fig. 1). Five treatments were compared that included single challenges with sublethal doses of fungi (F), bacteria (B) or imidacloprid (I) and dual challenges with fungi or bacteria + imidacloprid (F+I or B+I). Microarrays contained a mix of ~14,500 cDNA oligonucleotides representing ~10,500 host gut and protist/ symbiont genes, including stereotypical immune response genes [10, 20, 21], and thus provided simultaneous assessments of host and protist gene expression. Our findings point toward multiple modes of action through which insecticide-pathogen synergy happens and support the existence of novel routes through which nicotinoid insecticides and pathogens can interact to cause deleterious impacts on social insect colonies.

## Results

### Impacts on termite and symbiont behavior and survival

**Fig 2:**
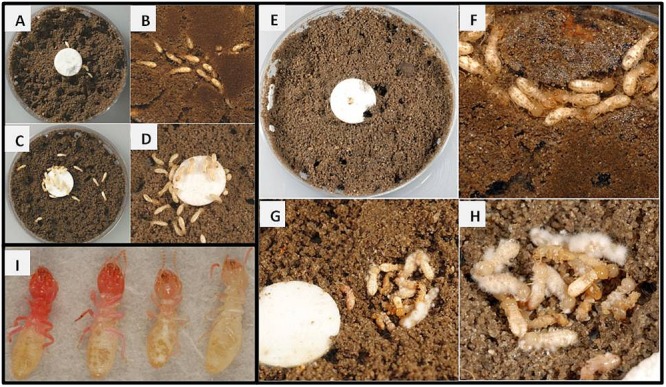
Representative examples of normal and infected termites. (A,B) Control termites displaying normal behaviors and (C,D) imidacloprid treated termites displaying mild intoxication effects. (E,F) Termites treated with sublethal fungal dose showing no effects, and (G,H) termites displaying mycosis after treatment with fungi + imidacloprid. (I) Termites treated with bacteria + imidacloprid showing varying degrees of Serratia infection.

**Fig 3:**
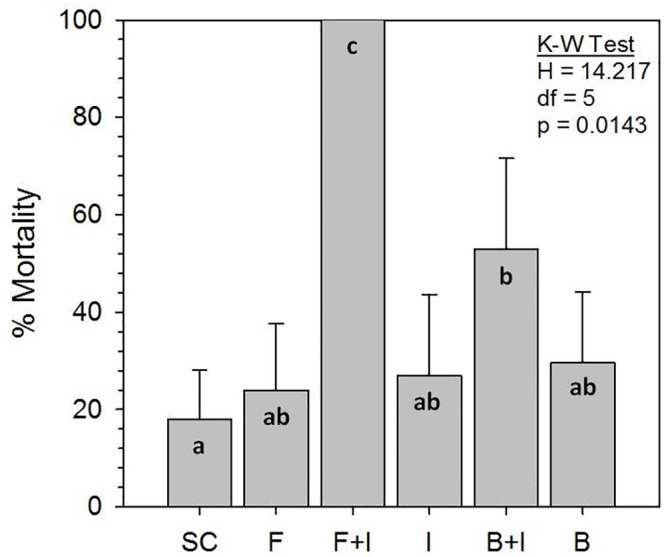
Bioassay mortality after 7 day exposures to six treatments. Highest mortality occurred in combination treatments of fungi + imidacloprid and bacteria + imidacloprid (see Fig. 1 for treatment abbreviations). Kruskal-Wallis (K-W) test statistics are shown indicating significance of the whole model. Bars with the same letters (a,b,c) are not different by Mann-Whitney U-tests (p<0.05).

Seemingly normal behaviors, *i*.*e*., grooming, tunneling, and light repellency, as well as no mortality, were displayed by solvent controls (SC) (Fig. 2A, B). Termites exposed to a sublethal imidacloprid concentration neither produced tunnels nor were repelled by light (Fig. 2C, D). Termites exposed to fungi or bacteria alone displayed normal behaviors (Fig. 2E, F), while those exposed to combined F+I or B+I treatments displayed clear pathology (Fig. 2G, H, I). Mortality at 2 days, the time at which termites were sampled for microarray assessment, was not different among the 6 treatments (*p* = 0.2609; Avg = 7.0%, range = 1–22%). By 7 days, however, imidacloprid greatly synergized the virulence of fungi, causing 100% mortality (Fig. 3), which is significantly greater than would be predicted by summation of the F and I single treatment mortalities (*p* = 0.0182). Mortality caused by the B+I combination was more than that from B or I alone (Fig. 3), but it was less than the F+I combination and was not synergistic (*p* = 0.5947).

We also were interested to determine if imidacloprid indirectly or directly impacted protist and bacteria symbiont populations. This question was addressed by *post-hoc* investigations of imidacloprid treatment on protist and bacterial gut symbiota. Regardless of the variation in initial densities, imidacloprid exposure reduced protist populations in 5 of 6 treated termite colonies (S1 Fig.). The morphology and motility of the protists from treated and control termites were similar. Treatment did not seem to target any specific protist clade but resulted in a ~25–50% reduction of representative protist groups. Conversely, based on two independent measures, gut bacterial populations were not affected by imidacloprid exposure. These independent bacterial measures included (i) quantitation based on culturable colony-forming units (S2A Fig.) and (ii) 16S rDNA abundance (S2B Fig.).

### General transcriptome level impacts

Microarray analyses were conducted for the purpose of identifying treatment impacts at the transcriptome level. Arrays contained ~14,500 cDNA oligonucleotides representing a blend of ~10,500 host gut and protist-symbiont genes, with positions annotated accordingly as being from host, symbiont, or mixed origins (see [10,20,21] for details). RNA of whole guts from 5 replicate colonies was sampled 2d post-exposure to each of the 6 treatments. Gene expression for the F, B, I, F+I or B+I exposed guts was normalized individually to the common solvent control (SC) treatment. Only positions changing by +/- 1.2-fold and *p*<0.05 were considered further. qRT-PCR was used to validate microarray results for a subset of 34 F+I passing genes, using the original F+I, F, and I cDNA samples as qRT-PCR templates. As with our prior microarray studies conducted in parallel with the current study [20,21], these validations revealed a significant correlation between microarray and qPCR results (S3 Fig.).

**Fig 4:**
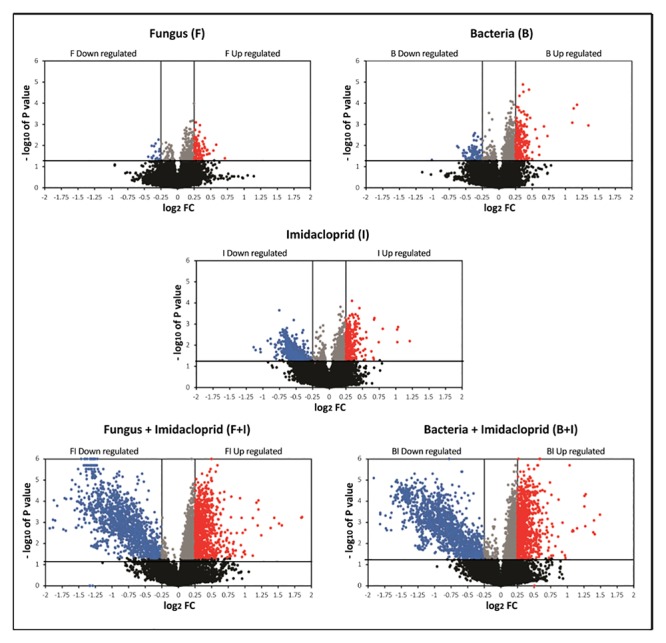
Microarray volcano plots showing differing numbers of passing array positions in F (fungi), I (imidacloprid), B (bacteria), F+I (fungus + imidacloprid), B+I (bacteria + imidacloprid) treatments. Red and blue spots indicate significant upregulated and downregulated array positions with +/- 2-fold change (FC) upregulation and downregulation, respectively.

In agreement with bioassay results (Fig. 3), microarray volcano plots show larger numbers of array positions responding in the dual F+I and B+I treatments compared with the single F, B, and I treatments (Fig. 4). Exposure to the bacterial or fungal propagules alone induced comparatively minor changes in the gut metatranscriptome. In comparison, exposure to sublethal concentrations of the nicotinoid (I) caused greater gene downregulation than did B and F treatments; likely, this was a result of the impact of this chemistry on the hindgut protist community (see above). Venn diagrams showing passing array positions shared among treatment categories are provided in S4 Fig. The F, B, and I treatments upregulated 85, 214, and 260 transcripts in the array, respectively, and downregulated 16, 89, and 504 array positions, respectively. Combining the B or F with I led to synergized increases in both upregulated and downregulated array positions. Overall, more array positions are shared among the F+I and B+I dual treatments than among the single F, B, and I treatments, indicating that imidacloprid plays a key role in altering gene expression.

Next, after forming sequence contigs at the 90% similarity level from only passing array positions, the host or symbiont origins of the contigs and remaining “singlets” were tallied across treatments (Table 1; S1–S5 Tables). In total, 3,187 genes were differentially (*p*<0.05) expressed across all comparisons, with 79% occurring in the F+I and B+I combination treatments. Among the single-challenge treatments, imidacloprid had the largest impact on transcript expression profile (393), followed by bacteria (196) and fungi (83). Finally, the majority of upregulated genes across all treatments were of host origin, and conversely, the majority of downregulated genes were of symbiont origin (Table 1).

**Table 1:** Summary table showing up- and downregulated contigs and “singletons” from host and symbiont in each treatment category (F, B, I, F+I and B+I).

	UPREGULATED				DOWNREGULATED				
	Host	Symbiont	mixed	Totals	Host	Symbiont	mixed	Totals	Overall
**F**	56	12	1	69	6	8	0	14	**83**
**B**	142	13	1	156	6	34	0	40	**196**
**I**	157	19	1	177	8	205	3	216	**393**
**F+I**	568	48	3	619	42	567	7	616	**1235**
**B+I**	634	63	4	701	26	545	8	579	**1280**
**Totals**	1557	155	10	1722	88	1359	18	1465	**3187**

Overall, greater numbers of host genes were upregulated and symbiont genes were downregulated by the various treatments. “Mixed” refers to genes that were sampled from both host and symbiont libraries.

### Gene ontology and pathway analyses

All passing contig and singleton sequences from microarrays were subject to analysis by KEGG and BLAST2GO (see Methods and Materials for details). First, KEGG analyses revealed impacts on general housekeeping pathways, mainly showing they are downregulated in I, F+I, and B+I treatments (S6–S10 Tables). These downregulated KEGG pathways include glycolysis and gluconeogenesis, the TCA cycle, purine and amino sugar metabolism, and others. We then conducted BLAST2GO analyses, which included the three gene ontology (GO) analyses *Molecular Function* (MF), *Biological Process* (BP), and *Cellular Location* (CL). Consistent with general results summarized in the preceding section, the paired F+I and B+I treatments had larger numbers of terms in all three GO categories than did the single treatments (S5 Fig.). Overall, the F+I treatment had more GO terms in each category, followed closely by B+I. Also, the majority of terms in the I and F+I treatments were downregulated (S11– S13 Tables). Top MF terms included hydrolase activity and various types of binding (*i*.*e*., nucleotide, protein, ATP, GTP, and broad-spectrum). Top BP terms included cellular and nucleobase-containing metabolic processes, transmembrane and intracellular protein transport, GTPase-mediated signal transduction, and anatomical structure morphogenesis. Top CL terms included intracellular, cytosol, cytoplasm, protein complex, ribosome, and membrane locations.

Because of the synergy observed with F+I treatments, this category is considered in finer detail. In the F+I category, the top upregulated MF terms all included binding (protein, ATP, zinc ion, and nucleotide), but the most downregulated terms also included binding (GTP, ATP, and protein). The top upregulated BP terms were proteolysis, oxidation-reduction and carbohydrate metabolic processes; the most downregulated were GTP catabolism, microtubule-based movement, and protein polymerization. In the CL category, the top upregulated terms were extracellular region, membrane, nucleus, and intracellular locations, whereas the most downregulated terms were cytoplasm, microtubule, and integral to membrane.

### Candidate Genes

A subset of 79 responsive candidate genes from 9 categories is summarized in Table 2. A complete summary of all 3,187 responsive genes across all treatments is provided in S1–S5 Tables. Categories considered in detail included *antimicrobial*, *carbohydrate-active*, *chemosensory*, *detoxification*, *JH-responsive*, *neuropeptide*, *cytochrome P450*, *transcription factor*, and “*other*.” Most antimicrobial genes from the candidate list were upregulated in either the F+I or B+I treatment (Table 2). Key antimicrobial genes identified include *lysozyme*, *PRPs*, *termicin*, *transferrins*, and *leucine-rich repeat proteins*. Many carbohydrate-active genes were differentially expressed among treatments, but the most notable are the *GHF7 cellulases*, which were all significantly downregulated in the F+I treatment, including one isoform that was downregulated over 1000x. Several of the same GHF7s downregulated in the F+I treatment were upregulated with the less impactful B+I treatment. Several chemosensory-related genes from the *takeout* family were also upregulated with various treatments and particularly in F+I.

**Table 2:** A subset of upregulated (values >1) and downregulated (values <1) candidate genes identified across all microarray treatment categories (F, B, I, F+I and B+I) and their origins from either host or symbiont (see text for details).

		Fold change (by treatment)					
Category	Candidate genes	F	B	I	F+I	B+I	Origin
**Antimicrobial**	antimicrobial peptide 7848				1.41		Host
	cathepsin b				0.423		Symbiont
	ferric-chelate reductase 1				1.34		Host
	heat shock protein				0.271		Symbiont
	heat shock protein				0.261	2.59	Symbiont
	heat shock protein 90					2.67	Symbiont
	laccase 2				1.37		Host
	lysozyme p				1.43		Host
	lysozyme precursor				1.67		Host+Symbiont
	peptidoglycan recognition partial				1.77		Host
	peptidoglycan-recognition protein s2				1.81		Host
	termicin				1.39		Host
	transferrin				1.31		Host
	transferrin 3				1.50		Symbiont
	alpha amylase				1.69		Host
	cell surface leucine-rich repeat-containing protein		1.23	1.25			Host
	leucine-rich repeat-containing protein			0.68		2.08	Symbiont
	leucine rich repeat family			0.74		1.46	Symbiont
	leucine-rich repeat-containing protein 48			0.73		1.88	Symbiont
	leucine rich repeat protein 1			0.64		1.44	Symbiont
	leucine rich repeat family protein			0.71		1.72	Symbiont
	leucine rich repeat family					1.49	Symbiont
	leucine-rich repeat-containing protein 56					1.74	Symbiont
	leucine rich repeat family					1.48	Symbiont
**Carbohydrate-active**	alpha—mannosyl-glycoprotein 2-beta-n-acetyl-glucosaminyltransferase	1.36					Host
	beta-galactosidase-like	1.33		1.29	1.48		Host
	GHF 1 (beta-glucosidase)			1.28	1.32		Symbiont
	beta-lactamase				0.550		Symbiont
	brain chitinase and chia				1.24		Host
	carbohydrate-binding protein				0.715		Host
	chitinase-like protein idgf4-like	1.19					Host
	c-type lectin precursor					0.66	Host
	dockerin			0.77	0.545	1.57	Symbiont
	endo—beta-d-glucanase				1.33		Symbiont
	GHF 3				0.466		Symbiont
	GHF 3 N-terminal domain protein			0.67		1.78	Symbiont
	GHF 10				0.733		Symbiont
	GHF 7		0.80	0.76	0.001	1.61	Symbiont
	GHF 7				0.480	1.71	Symbiont
	GHF 7				0.572	1.51	Symbiont
	GHF 7				0.552	1.51	Symbiont
	GHF 7				0.508		Symbiont
	GHF 7				0.832		Symbiont
	GHF 7				0.786		Symbiont
	GHF 7				0.536		Symbiont
	GHF 13 (maltase 2)	1.22					Host
	maltase a2	1.26					Host
**Chemo-sensory**	takeout family protein		1.20	1.21			Host
	takeout family protein			1.20			Host
	takeout family protein				1.23		Host
	takeout family protein				1.20		Host
	takeout family protein(JHBP like)				1.25		Host
**Detoxification**	abc transporter family protein				0.551	1.54	Symbiont
	abc transporter family protein				0.545	1.68	Symbiont
	catalase			1.20			Host
	epoxide hydrolase 4-like					0.81	Host
	multidrug resistance protein 2					2.46	Symbiont
	peroxidase ppod1					2.39	Symbiont
**JH-Responsive**	50 Kda midgut protein		2.09	1.95	2.30		Host
	insulin receptor				1.36	1.29	Host
	JH-inducible protein		1.50	1.46			Host
	nli interacting factor-like phosphatase family protein				2.37	0.36	Host
	tyramine beta hydroxylase				1.42		Host
	arylsulfatase j-like			1.24			Host
**Neuropeptide**	allatostatin neuropeptide precursor	1.27			1.50		Host
	neuropeptide f				1.26		Host
**Cytochrome P450 (CYP)**	CYP304A1-like		1.23		1.43		Host
	CYP4C1-like		1.24	1.24	1.25		Host
	Cyp4C1-like			1.21	1.25		Host
	CYP6AM1-like		1.26	1.27	1.35		Host
	CYP6K1-like				1.22		Host
	CYP9E2-like			1.30	1.34		Host
	CYP9E2-like				1.20		Host
	CYP15F1 (*R*. *flavipes*)			1.19			Host
	CYP4U3V1 (*R*. *flavipes*)				1.22		Host
**Transcription factor**	EF hand family protein					2.48	Symbiont
	fork head				1.22		Host
	RNA-binding protein luc7-like 2-like		1.60	1.47			Host
**Other**	cysteine synthase a			0.46	0.301		Symbiont

See S1–S5 Tables for a full listing of significant responsive genes from each treatment category.

Two inter-related groups are the detoxification and P450 categories. Key detoxification genes included *ABC transporters* (downregulated in F+I; upregulated in B+I), *catalase*, and *epoxide hydrolase*. Nine P450s from the *CYP4*, *6*, *9*, *15*, and *304* families were all upregulated in response to various treatments, but mostly in the F+I treatment. Several genes occurring in the JH-responsive category were initially identified in a prior study specifically investigating JH impacts on caste differentiation and gut gene expression: *50kDa Midgut protein*, *insulin receptor*, *nli phosphatase*, *tyramine beta hydroxylase*, and *arylsulfatase*. Two neuropeptide-encoding genes were also upregulated that included *allatostatin* and *neuropeptide F*. Three transcription factors were all upregulated with I, F+I and/or B+I treatments; one (*EF Hand family protein*) was previously identified in association with dietary phenolics and potentially phenolic-mediated melanization processes. Lastly, in the “other” category, a gene that was significantly downregulated in I and F+I treatments (*cysteine synthase a*) was previously upregulated by cellulose feeding (the substrate used in the current study) [20].

## Discussion

### Overview

The present study builds on three prior studies that used the same termite colonies to investigate nutritional, hormonal and social impacts on gut metatranscriptome composition [20,21] and characterize gut microbiota composition and its recalcitrance to dietary changes [16]. Findings of the present study point toward multiple modes of action through which insecticide-pathogen synergy occurs. Our findings suggest mechanisms through which nicotinoid insecticides and entomopathogens can interact to cause deleterious impacts on social insect colonies. Nicotinoids such as imidacloprid disrupt the insect nervous system by agonizing nicotinic acetylcholine receptors, leading to excessive neuroexcitation and eventually, irreversible neurological disruption [22]. Thus, a plausible explanation for nicotinoid-fungal synergy is disruption of hygienic behaviors by nicotinoid action [3,24]. As a soil termiticide, imidacloprid has unique physical properties that allow it to be acquired, move among individual termites via trophallaxis and contact, and eventually affect colonies at substantial distances away from treated structures [17]. Nicotinoids taken up by termites are rapidly metabolized to a mix of active and inactive metabolites, most notably, glucuronic acid conjugates [23]. Their formation is noteworthy because of the availability of both glucose and glucuronic acid in termite food (*i*.*e*., cellulose and hemicellulose) and the potential for such conjugate formation to be mediated by hindgut symbiont action. These conjugates are also highly water-soluble and more likely to be transferrable by trophallaxis and allogrooming. Thus, imidacloprid toxicokinetics, transfer, and neurological disruption are all factors to consider in relation to key study outcomes as discussed below.

### Effects at the Organismal and Sub-organismal Levels

The addition of sublethal concentrations of imidacloprid in all treatments suppressed many of the termite’s social behaviors (grooming, trophallaxis, and tunnel formation) that protect these soil-dwelling insects from disease [3]. The impacts of sublethal imidacloprid concentrations on termite tunneling and grooming behaviors can be observed within 24 h post-exposure. In F treatments, grooming of colony conspecifics results in the near-complete removal of conidiospores attached to the cuticle within 24 h; the ingestion of these conidiospores by groomers places the fungal propagules in contact with a gut microbiota that is apparently highly antagonistic/ suppressive to potential pathogens. Similarly, tunnel formation by termites is also highly antagonistic to microbial growth; termites coat the tunnels with the gut microflora and metabolites that presumably suppress or outcompete pathogens for available resources [3,25].

Recently, nicotinoids have been reported to suppress the innate immune response in several insects. For example, Di Prisco *et al*. [26] reported in honey bees that the neonicotinoid clothianidin upregulates a leucine-rich peptide that is a negative modulator of the nuclear factor-kβ signaling. The result of this immunosuppression is increased replication of the deformed wing virus in covertly infected honeybees. The downregulation of nuclear factor-kβ signaling may also influence gut microbiota homeostasis as observed in *Drosophila* [27] and, therefore, disrupt nutrient digestion and nutrient assimilation [26]. In our study, imidacloprid treatment upregulated a transcript that was annotated as a host cell membrane leucine-rich peptide, but it failed to alter expression of other genes stereotypically associated with the innate immune response (Table 2). These findings agree with the previous finding in *R*. *flavipes* that exposure to sublethal doses of imidacloprid failed to alter its phagocytic response to nonself [3].

An additional consequence of imidacloprid treatment was a decrease in hindgut protist symbiont populations (S1 Fig.). There are no prior reports of imidacloprid being active against eukaryotic protists, but nicotine-like substances and their analogues are known to have antibacterial activity in other systems [28]. However, assays testing imidacloprid-impregnated paper discs at concentrations 1-1000-fold higher than used in our feeding assays did not suppress the growth of the aerobic, culturable bacterial community from the *R*. *flavipes* digestive tract (S2A Fig.). These assays did not examine imidacloprid’s impact on the anaerobic and/or unculturable bacteria comprising the majority of the gut microbiota [16], but investigation of total bacterial DNA abundance using 16S PCR signals as a proxy did not detect impacts in imidacloprid-treated individuals (S2B Fig.). Potentially, sublethal concentrations of imidacloprid could indirectly alter bacteria-host associations through a reduction in feeding or trophallaxis and modify the suitability of the hindgut as a protozoan microhabitat. The protists that remained in the gut, although significantly fewer in number, were observed to be alive. The reduction in protist numbers is reflected in the selective downregulation of symbiont genes in all treatments that included imidacloprid. For example, exposure to imidacloprid (I) alone upregulated a majority of host transcripts (177 host vs. 19 symbiont), but downregulated a majority of protist symbiont transcripts (205 symbiont vs. 8 host). Also, protist GHF7 cellulases were substantially downregulated in the F+I treatment, suggesting important digestion-immunity tradeoffs (see below).

### Immune Gene Expression

Cuticular exposure or ingestion of fungi (F) or bacteria (B) did not infect or cause a lethal mycosis or sepsis in treated *R*. *flavipes*. Unlike the I treatment, exposure to fungal and bacterial entomopathogens did not alter the termite eusocial behaviors that underlie pathogen resistance. The presence of the fungus on the cuticle and then in the gut (via grooming) induced the lowest number of transcript changes seen in the study. Cuticular exposure to a high concentration of conidiospores upregulated significantly more host (56) than symbiont transcripts (12) but downregulated low numbers of both symbiont (8) and host (6) transcripts. Thus, ingestion of fungal spores via grooming elicited only minor changes in gut symbiont populations. Both treatments resulted in minor fold changes in both upregulated (69) and downregulated (14) host and symbiont transcripts. Ingestion of bacteria stimulated more transcripts to be altered at a greater fold change than the F treatment, and like the F treatment, more transcripts were upregulated in the B treatment (142 host, 13 symbiont) than were downregulated (6 host, 34 symbiont).

Apparently, elicitors associated with the ingested bacteria, although capable of upregulating host transcripts, are not antagonistic to gut protists that co-inhabit the hindgut with a complex high-density bacterial community [16]. Microarrays included ~40 antimicrobial host genes that were annotated as components of the phenoloxidase cascade, various nonself recognition proteins (lectins, GNBPs, and chitin-, LPS- and glycan-binding peptides), cationic peptides, programmed cell death proteins, and enzymes (serpins). Treating termites orally with bacterial cells or topically with fungal conidiospores caused no significant alteration in the transcription of these defense-related genes. This lack of detectable upregulation of gut-associated innate defense genes by the bacterial challenges was unexpected. *Serratia marcescens* is a well-known opportunistic insect pathogen [29] and, like other Gram-negative opportunistic bacteria [30,31], is known to elicit insect gut innate defenses [32,33].

As observed previously, the consequence of exposure to sublethal imidacloprid concentrations is the rapid onset and complete mycosis by normally ineffective entomopathogenic fungi [3]. Termites exposed to the F+I treatment at two days were presumed to contain replicating vegetative *M*. *anisopliae* cells at the time of sampling, as more than 70% of the insects alive at 2 days (*i*.*e*., the point of RNA extraction for gene expression studies) succumbed to mycosis by 3 days post-challenge. The combination of B+I also led to increased (but not synergistic) levels of sepsis. Unlike the F or B treatments, the F+I treatment, which resulted in lethal mycosis, upregulated several antimicrobial host genes, including *lysozyme*, *PRPs*, *termicin*, and *transferrins*. These findings suggest that imidacloprid, rather than suppressing physiological immune mechanisms, instead blocks immune-related behaviors and allows the fungus to invade the host. The ensuing pathogen ingress then elicits the termite’s innate defense response, which is incapable of preventing a lethal mycosis.

The B+I treatment resulted in 50% lethal sepsis and also upregulated two symbiont heat-shock proteins; however, like the B treatment, the B+I treatment caused no significant alterations in termite gut antimicrobial transcripts. Conversely, in the termite *Coptotermes formosanus*, subtracted mRNA libraries from whole insects revealed that microbial challenge (topical exposure) upregulated a cascade of immune-associated genes [11]. Another study in *C*. *formosanus* comparing candidate gene expression responses to xenobiotic and bacterial challenges similarly identified an induction of immune and xenobiotic response genes [34]. The inability of sepsis to induce gut innate defenses as seen here may be due to the lack of inducible genes being present on the microarray, improper timing in the sampling of gut mRNA, or weak innate defenses in these social insects. For example, the arrayed target genes were all derived from ESTs generated from gut mRNA of healthy workers [10]. The termites sampled in the present study were orally challenged with a single bacterial strain and only gut mRNAs (not fat body) were sampled at a single interval. Possibly, using different bacteria, cell concentrations, or sampling different tissues at additional time intervals would have shown upregulation of the antimicrobial genes. However, previous research on *R*. *flavipes* has shown that injection of LPS, a universal elicitor of insect innate defense pathways, fails to induce the synthesis of cationic peptides, further supporting the idea that this species possesses a weak innate defense system. These findings might suggest that *R*. *flavipes* relies on a combination of hygienic behaviors and gut microbial ecology to create a microclimate that is antagonistic to potential entomopathogens.

### Expression of non-immune genes

In terms of non-immune-related genes, many carbohydrate-active genes were differentially expressed among treatments. The most notable are the symbiont *GHF7 cellulases*, which were all significantly downregulated in the F+I treatment, including one isoform that was downregulated over 1000x. Conversely, several GHF7s downregulated in the F+I treatment were upregulated with the less-lethal B+I treatment. None of the GHF7s identified here were responsive in preceding diet or hormonal microarray studies [20,21]. Several chemosensory-related genes from the *takeout* family were upregulated with various treatments, particularly F+I. These genes are relevant to chemical communication [35] that potentially directs eusocial disease management.

Two additional inter-related categories are detoxification and P450 genes. Key responsive detoxification genes included *ABC transporters*, *catalase*, *epoxide hydrolase*, and *P450*s. In particular, two ABC transporters were downregulated in F+I and upregulated in B+I treatments. Nine P450s from the *CYP4*, *6*, *9*, *15*, and *304* families were all upregulated in response to various treatments, but mainly to F+I. Similar *CYP15* responses were also documented in *C*. *formosanus* workers in response to bacterial and xenobiotic challenges [34]. Also, several genes occurring in the JH-responsive category were initially identified in a prior study specifically investigating JH impacts on caste differentiation and gut gene expression: *50kDa Midgut protein*, *insulin receptor*, *nli phosphatase*, *tyramine beta hydroxylase*, and *arylsulfatase*. Of these, the *50kDa* gene was the most JH-responsive, but it has no GO terms and few homologues in other insects [21]. Most of these genes were upregulated in the various treatments, suggesting parallels between JH-induced morphogenesis and gut restructuring as a mechanism of pathogen defense.

Two neuropeptide-encoding genes also were most highly upregulated in the F+I treatment: *allatostatin* and *neuropeptide F*. Allatostatins regulate JH biosynthesis [36] and neuropeptide F controls gut peristalsis [37]; both processes potentially mediate pathogen defense. Three transcription factors were all upregulated with I, F+I and/or B+I treatments; one (*EF Hand family protein*) was previously identified in association with dietary phenolics and potentially phenolic-mediated melanization processes [38]. Lastly, in the “other” category, a protist *cysteine synthase*, a gene that was significantly downregulated in the I and F+I treatments, was previously upregulated by cellulose feeding (the substrate used in the current study [20]).

The major themes emerging from this examination of select candidate genes include digestion-immunity tradeoffs, gut remodeling, gut physiology, and social behavior as hallmarks and potential mechanisms of nicotinoid-pathogen synergy. Particularly important driving factors appear to be the significant degree of symbiosis occurring in the *R*. *flavipes* gut (*i*.*e*., 11 protists and >5000 bacterial OTUs), the susceptibility of protists to imidacloprid (Fig. 4), and the possible suppression of the host immune response in order to protect bacterial symbiont populations and to preserve an appropriate ecological balance in the hindgut.

### Conclusions

This study took a microarray-based approach to gain new molecular-level insights into mechanisms of nicotinoid-pathogen synergy. Termites are a relevant model for this work because they are eusocial insects that maintain complex microbial symbioses, and they are purposely targeted by nicotinoid soil termiticides. We tested three hypotheses regarding the mechanistic underpinnings of nicotinoid-pathogen synergy: that imidacloprid suppresses (1) hygienic social behaviors, (2) gut symbiont populations, and/or (3) components of the innate immune response. Our findings strongly support the first two hypotheses, *i*.*e*., that compromised social behaviors and nicotinoid-dependent reductions in protist symbiont populations underlie nicotinoid-pathogen synergy. Very little molecular/ transcriptomic evidence was found in support of the third hypothesis relating to a compromised immune response in the gut, and likewise, no impacts by imidacloprid on gut bacterial populations could be identified.

As our work did not consider gene expression outside the gut, investigating the immune response outside the gut seems necessary to provide better resolution. Alternatively, the decreases in protist abundance and gene expression we observed after sublethal imidacloprid exposure may be key factors promoting fungal pathogenesis. In particular, the significant suppression of several protist GHF7 cellulases, which are important to digestion [20,38,39] may also promote fungal susceptibility. This finding is consistent with protist declines that were independently verified, and it suggests possible overlap of nutritive and immune functions by this enzyme class, or possibly energetic tradeoffs between lignocellulose digestion and immune function [40]. Conversely, the less lethal impacts by the *Serratia* bacterial entomopathogen tested may be linked to the large and diverse population of bacterial symbionts present in the *R*. *flavipes* gut [16]. Possibly, the maintenance of bacterial symbionts in an ecological equilibrium (*i*.*e*., *symbiont-mediated immunity*) might be an evolved mechanism that serves to out-compete invading bacteria, rather than relying on more non-specific host immune mechanisms.

Finally, other possibilities suggested by this work include upregulation of chemosensory genes as a physiological basis for behavior-based eusocial disease management, endocrine-linked gut remodeling as a component of pathogen defense, and compromised xenobiotic responses as components of nicotinoid exposure and nicotinoid-dependent pathogenesis. Thus, our findings point toward a combination of diverse symbiont- and host-linked mechanisms for both pathogen defense and nicotinoid-pathogen synergy. These findings significantly advance our understanding of antimicrobial defenses in this important social insect group, and additionally, they provide novel insights into how nicotinoids may exert deleterious effects on social insect colonies.

## Methods and Materials

### Termites

Worker termites were used exclusively. Five established laboratory colonies isolated from field sites near Gainesville, FL, USA were used: *B1#1* (1 year in the lab); *B2* (3 months in the lab); *K2* (3 years in the lab); *K5* (2 years in the lab); and *K9* (3 months in the lab). All colonies were verified as *R*. *flavipes* by mitochondrial 16S rRNA sequencing [41]. Colonies were maintained in darkness in sealed plastic boxes with wet pine wood shims and brown paper towel, within an environmental chamber kept at 22°C and 60% RH. Preceding studies on three colonies (B2, K5 and K9) showed them to have significantly variable bacterial microbiota compositions that are recalcitrant to change under different 7d dietary regimes [16], whereas the host and protist gut gene expression profiles of all five colonies responded significantly to dietary, hormonal, and social treatments [20,21].

### Pathogen bioassays

Separate assays were performed for microarray analysis and for assessing survivorship. For immune challenges, concentrations of fungal spores, bacterial cells, and imidacloprid were selected based on published findings [3,42,43] and on results from preliminary screening assays. For fungal treatments, spores of *Metharizium anisopliae* (isolate Ma1630) were collected from *in vitro* cultures 10–12 d after inoculation onto McCoy’s agar, suspended in 0.5% of aqueous Tween 20, counted in a hemacytometer, and diluted with water to a final concentration of 10^5^ spores/ml. Each replicate of 20 termites was placed in a steel mesh specimen basket (16 mm outer diameter, 8 mm high) and submerged in 5 ml of spore suspension for 20s. After removal of excess liquid with tissue paper, termites were gently tapped into the Petri dish. Viability of fungal spores was ≥94% and determined by spreading diluted aliquots of each suspension onto McCoy’s agar and recording germination after 24 h. For bacterial challenge, *Serratia marcescens* cells (isolate “New Zealand May 18”) were harvested from nutrient broth cultures during exponential growth phase and centrifuged at 5,900×*g* for 10 min at 4°C. Broth was removed, cells were suspended in sterilized saline (0.85% NaCl), and cell concentration estimated spectrophotometrically (OD600) and adjusted to 6.5 x 10^9^ cells/ml before 150μl was applied to filter paper discs (final dosage = 2.35 x 10^8^ cells/cm^2^), which served as food substrate. Viability of bacterial cells was confirmed by spotting diluted aliquots of cell suspensions onto nutrient agar and counting colony-forming units (CFUs) after 24 h. For insecticide treatments, filter paper discs were treated with a 0.0001% aqueous solution of imidacloprid (97.5% purity, Bayer, Pittsburgh, PA; initially dissolved at 1% w/v in dimethyl sulfoxide, DMSO) and allowed to air-dry before use. Termites in dual treatments were exposed to imidacloprid and either and *M*. *anisopliae* or *S*. *marcescens*. Discs for control treatments were pretreated with 0.0001% aqueous DMSO and moistened with 150μl saline.

### Protist and bacterial counts

*Post-hoc* tests were performed to further investigate imidacloprid impacts on protist and bacterial gut symbiota using multiple independent termite colonies. Protist counts were made using five laboratory colonies as described previously [44]. Bacterial CFU counts were done via aerobic culturing using two laboratory colonies; one that had been in the lab for >2 yr. (colony 1) and one collected from the field 1 month earlier (colony 2). Groups of termites from two colonies were exposed to imidacloprid or to solvent control treatments for 48 h. Whole guts dissected from individual termites were sonicated in 250 μL of PBS, serially diluted, and spotted (2 μL) onto nutrient agar plates. After incubation at 26°C for 24 h, plates were examined; spots producing 3–10 CFUs were used to estimate the total number of aerobic culturable bacteria per termite. In addition, dilutions of gut homogenates were directly plated onto nutrient agar; discs loaded with serial dilutions of imidacloprid were added to these plates to examine its direct impact on the culturable bacteria. Quantitative polymerase chain reaction (qPCR) was performed to determine bacterial abundance in each sample used for CFU counts, *i*.*e*., colonies 1 and 2, with and without imidacloprid treatment. DNA was isolated from termite whole guts following control or imidacloprid treatments in bioassays using the Epicentre Yeast DNA extraction kit, including RNase treatment (Madison, WI). Following isolation, DNA samples were subjected to phenol-chloroform cleanup and concentrated using sodium acetate-ethanol precipitation. qPCR reactions were performed in triplicate with SensiFast SYBR No ROX kit (Bioline; Taunton, MA), 50ng of sample DNA, nuclease-free water, and degenerate 16S rDNA primers. Primers amplified a 291 bp fragment containing the V4 hypervariable region of the 16S rRNA gene (U515F-GTGCCAGCMGCCGCGGTAA; U806R- 5’- GGACTACHVGGGTWTCTAAT-3’) [45]. Host DNA was quantified using primers specific to an apparent single-copy host gene, Actin 5C-1 (Act5CF-TCTGGTAGGACCACTGGTAT; Act5CR-GTATCCACGCTCCGTCAAA). Data were normalized to Actin 5C-1 to determine the relative abundance of 16S amplicons in the control and imidacloprid-treated DNA preparations.

### Gut extraction and RNA isolation

After two days, a subset of ten termites was removed from each of the replicate colony treatments (20 total samples), cold-immobilized, surface-sterilized by a serial rinse in 0.3% sodium hypochlorite (1x) and sterilized water (2x), and dissected on Parafilm to collect digestive tracts, including salivary glands. Digestive tracts were transferred into RLA Lysis Buffer (Promega, Fitchburg, WI, USA) and stored at -70°C until RNA isolation. RNA extraction and cDNA synthesis was done as described in preceding reports (Raychoudhury *et al*. [20], Sen *et al*. [21]).

### Microarrays and hybridization protocols

Experiments were designed after MIAME guidelines. A type II microarray [46] design was used with a common-reference strategy [47]. The common reference consisted of a normalized blend of all RNA samples included in the experiment. This common reference was co-hybridized against each replicate sample on single microarrays. Dye swaps [46, 47] were performed between replicate samples and references to check for potential dye impacts on spot intensity. Twenty-five total microarray hybridizations were performed, which consisted of five colonies each treated with solvent controls (SC) or sublethal doses of fungi (F), bacteria (B) or imidacloprid (I), and dual challenges with fungi or bacteria + imidacloprid (F+I or B+I). Microarray data are provided in S15–S20 Tables. These data are shown by array position for each treatment, normalized to mixed reference hybridizations, and include negative and positive controls. Genbank accession numbers for sequences at each microarray position are provided in S21–S23 Tables.

### Microarray statistical analyses

The Matlab statistics toolbox was used for statistical analysis of the intensity data of the 25 hybridizations from five different treatments (SC, F, B, I, F+I or B+I). Before comparative analysis, the individual signal intensity values obtained from the microarray probes were log-transformed (using 2 as the base) and normalized among all individual samples included in the study. Normalization was accomplished by scaling the individual log-transformed signal intensities so that each dataset had comparable lower, median, and upper quartile values [48]. After the data were normalized, Student’s t-tests were used to make probe-by-probe comparisons among treatments. In each comparison, a *p*-value and fold change were computed for all microarray loci. In addition to *p*-values, *q*-values were computed [49]. While the *p*-value measures the minimum statistical false-positive rate incurred when setting a threshold for test significance, the *q*-value measures the minimum false-discovery rate incurred when calling that test significant [49]. A volcano plot for each comparison was generated that displays the negative log_10_-transformed *p*-value versus log_2_-transformed fold change for each array locus.

### Bioinformatic analyses

For contig generation, all significantly differentially expressed array positions that met the fold-change criteria in each bioassay were selected and processed through Sequencher (Gene Codes Corporation, Ann Arbor, MI) with a minimum match percentage of 95 to generate contigs. The generated contigs and the remaining orphan sequences were used for further analyses using the program BLAST2GO [50] for identification and annotation. By using the inbuilt BLASTx algorithm, these sequences were used as queries in BLASTx searches against the Genbank non-redundant (nr) database with an *e*-value cut-off of ≤ 1e-03. The putative identification, annotation, and Gene Ontology (GO) terms [51] for the sequences also were obtained through BLAST2GO. KEGG analyses were performed as described previously [21,52].

### Validation of microarray fold-change data by quantitative real-time PCR

The fold-change data from the microarray results were validated by performing sets of quantitative real-time PCRs (qRT-PCR) with a CFX-96 Real-time System (Bio-Rad, Hercules, CA) using the SYBR-green detection method (SensiMix SYBR & Fluorescein one-step PCR reagent; Bioline, Taunton, MA). Thirty-four fungal-associated sequences (S6 Table) with varying degrees of fold change were used to design primer sequences using the web-based tool Real-time Design (http://www.biosearchtech.com/realtimedesign). The housekeeping gene *lim-1* was used as a reference gene [20,53]. Two μl of total RNA (from aliquots of 10 ng/μl) were taken from the original mRNA pools used for microarray hybridizations from all five colonies (5 treatments each) to synthesize cDNA using the iScript cDNA kit (Bio-Rad, Hercules, CA). Triplicate qRT-PCR reactions were performed for each of the biological replicate cDNA samples, along with a no-cDNA negative control, across the 34 primer sets (S14 Table). Cycling conditions were an initial step of 95°C for 3 minutes followed by 39 cycles of 95°C for 20 seconds, 56°C for 45 seconds, and 68°C for 50 seconds. Quantification was performed by first generating a standard curve of primer amplification efficiency using whole-gut cDNA from colony #1 with a five-fold dilution series and then extrapolating the experimental samples onto the curve. Each triplicate sample was averaged to one data point for ease of graphical representation. The mean delta threshold cycle (ΔC_T_) was calculated for each data point by subtracting it from the average C_T_ values of *lim-1*. Then, a ΔΔC_T_ value was calculated by subtracting average control (C) data points from F, B, I, FI, and BI treatments (see formula below using *F* as an example). These ΔΔC_T_ values were plotted against the corresponding fold- change levels from the microarray studies, and their associations determined non-parametrically by the Spearman rank correlation test.

$$\Delta \Delta {\text{C}}_{\text{T}}=\frac{1}{5}\overset{5}{\underset{j=1}{\sum }}(\frac{1}{3}\overset{3}{\underset{i}{\sum }}PF_{i}-\frac{1}{3}\overset{3}{\underset{i=1}{\sum }}lim1F_{i})-\frac{1}{5}\overset{5}{\underset{j=1}{\sum }}(\frac{1}{3}\overset{3}{\underset{i=1}{\sum }}PC_{i}-\frac{1}{3}\overset{3}{\underset{i=1}{\sum }}lim1F_{i})$$

j = number of biological replicates,

i = number of technical replicates,

P = given primer, lim1 = lim1 primer;

F_i_ = C_T_ value of the ith technical replicate from the fungal-treated termite gut cDNA,

C_i_ = C_T_ value of the ith technical replicate from the control treated termite gut cDNA

## Supporting Information

S1 Fig Imidacloprid impacts on protist survival. Protist survival counts 2 days (48 hr) after imidacloprid treatments in five separate termite colonies (B1, B2, W2, W3, W4, and W5). Black bars represent DMSO solvent controls and gray bars show imidacloprid treatments. Paired bars with asterisks (*) are not different by Mann-Whitney U-tests at different significance levels, as shown.
(JPG)

S2 Fig Imidacloprid impacts on gut bacterial numbers. Assessment of imidacloprid impacts after 48 hr on gut bacteria by two different assessment methods of (A) aerobic culturing and (B) 16S real-time quantitative PCR to estimate relative bacterial DNA abundance. Two colonies were used, one in the lab for >2 yrs. (colony 1) and one in the lab for <2 months (colony 2). Bars having the same letters for each colony within graphs are not significantly different by Mann-Whitney U-tests (p>0.05).
(JPG)

S3 Fig Correlations between microarray and qPCR fold changes for select candidate genes. Correlation between microarray fold-change (FC) and qRT-PCR fold-change (2-ddCT) values. Genes tested represented a subset of 35 array-positives from F+I treatments to verify the robustness of microarray results. A statistically significant correlation was found for FI and I treatments (B and C), but not F treatments (A), as expected.
(JPG)

S4 Fig Venn diagrams showing significant array positions shared among treatments. Venn diagram showing common array positions in 2-way (A) and 3-way comparisons involving FI (B) and BI (C) treatments. Paired FI and BI treatments shared many more positions in common than did single F, B, or I treatments.
(JPG)

S5 Fig Total numbers of up- and downregulated GO terms across treatments. Total numbers of upregulated (black) and downregulated (gray) GO terms across the treatment categories F, B, I, F+I and B+I, in the GO categories of Molecular Function (A), Biological Process (B) and Cellular Location (C). Greater numbers of GO terms occurred in the paired F+I and B+I treatments than in the single F, B, or I treatments.
(JPG)

S1 Table Summary of passing gene contigs in fungal (F) treatments. (XLSX)

S2 Table Summary of passing gene contigs in bacterial (B) treatments. (XLSX)

S3 Table Summary of passing gene contigs in imidacloprid (I) treatments. (XLSX)

S4 Table Summary of passing gene contigs in fungal + imidacloprid (F+I) treatments. (XLSX)

S5 Table Summary of passing gene contigs in bacterial + imidacloprid (B+I) treatments. (XLSX)

S6 Table KEGG terms for passing genes in the Fungal (F) array. (XLSX)

S7 Table KEGG terms for passing genes in the Bacterial (B) array. (XLSX)

S8 Table KEGG terms for passing genes in the Imidacloprid (I) array. (XLSX)

S9 Table KEGG terms for passing genes in the Fungal + Imidacloprid (F+I) array. (XLSX)

S10 Table KEGG terms for passing genes in the Bacterial + Imidacloprid (B+I) array. (XLSX)

S11 Table Molecular function (MF)—Blast2GO summaries for passing gene contigs. (XLSX)

S12 Table Biological process (BP)—Blast2GO summaries for passing gene contigs. (XLSX)

S13 Table Cellular location (CL)—Blast2GO summaries for passing gene contigs. (XLSX)

S14 Table Sequences for primers used in qRT-PCR validations. (XLSX)

S15 Table Microarray data for DMSO solvent (S) control treatments. (XLSX)

S16 Table Microarray data for fungi (F) treatments. (XLSX)

S17 Table Microarray data for bacteria (B) treatments. (XLSX)

S18 Table Microarray data for imidacloprid (I) treatments. (XLSX)

S19 Table Microarray data for fungi + imidacloprid (F+I) treatments. (XLSX)

S20 Table Microarray data for bacteria + imidacloprid (B+I) treatments. (XLSX)

S21 Table Genbank accession numbers for microarray positions having TG designations. (TXT)

S22 Table Genbank accession numbers for microarray positions having TS designations. (TXT)

S23 Table Sequences for microarray positions having “Random” designations. (TXT)
