# Supplementary figures and images for: Molecular Signatures of Nicotinoid-Pathogen Synergy in the Termite Gut
Source: PLoS One. 2015 Apr 2;10(4):e0123391. doi: 10.1371/journal.pone.0123391 (PMC4383478; doi:10.1371/journal.pone.0123391)

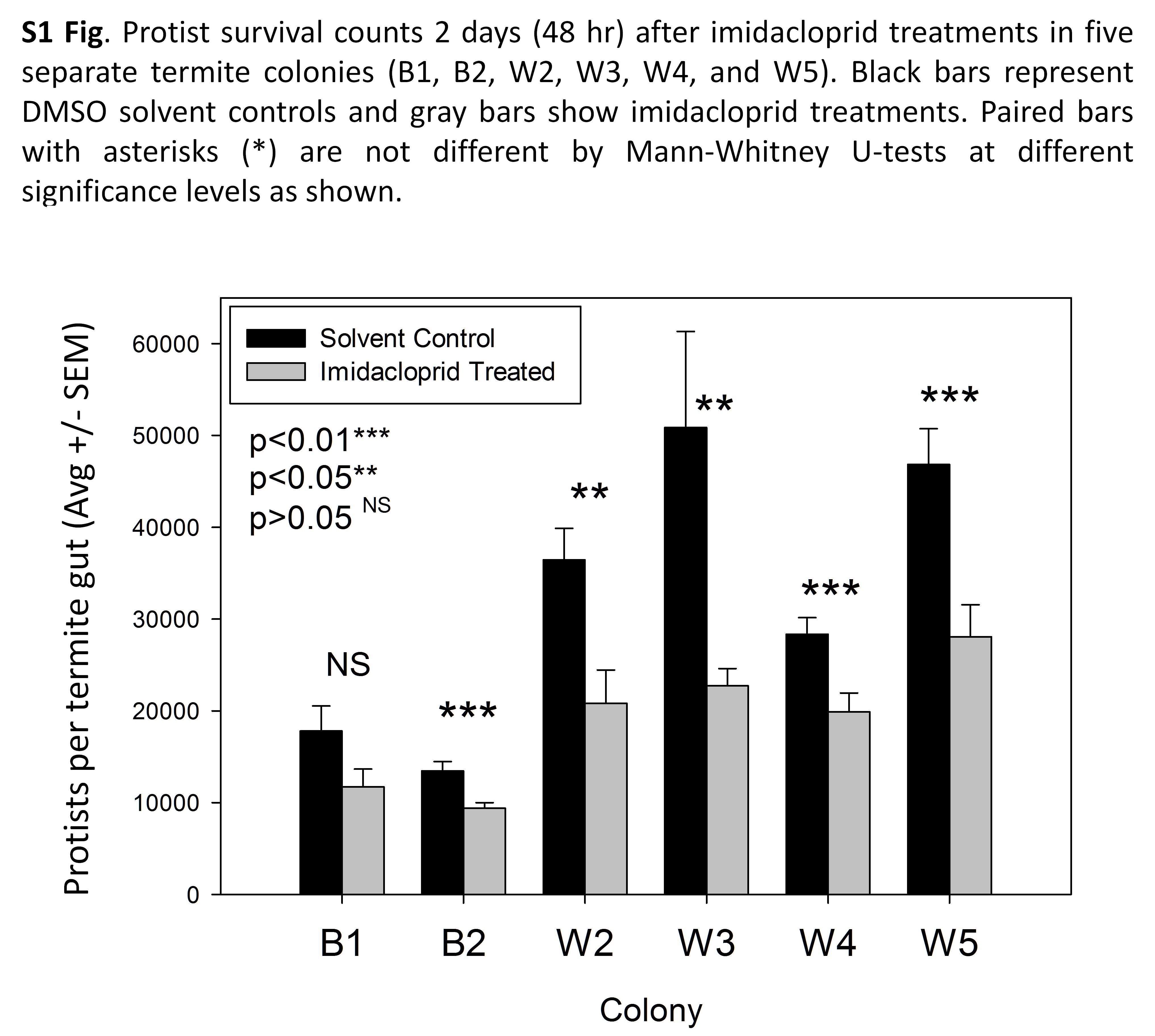

Supplement: S1 Fig — Protist survival counts 2 days (48 hr) after imidacloprid treatments in five separate termite colonies (B1, B2, W2, W3, W4, and W5). Black bars represent DMSO solvent controls and gray bars show imidacloprid treatments. Paired bars with asterisks (*) are not different by Mann-Whitney U-tests at different significance levels, as shown. (JPG) [file pone.0123391.s001.jpg]

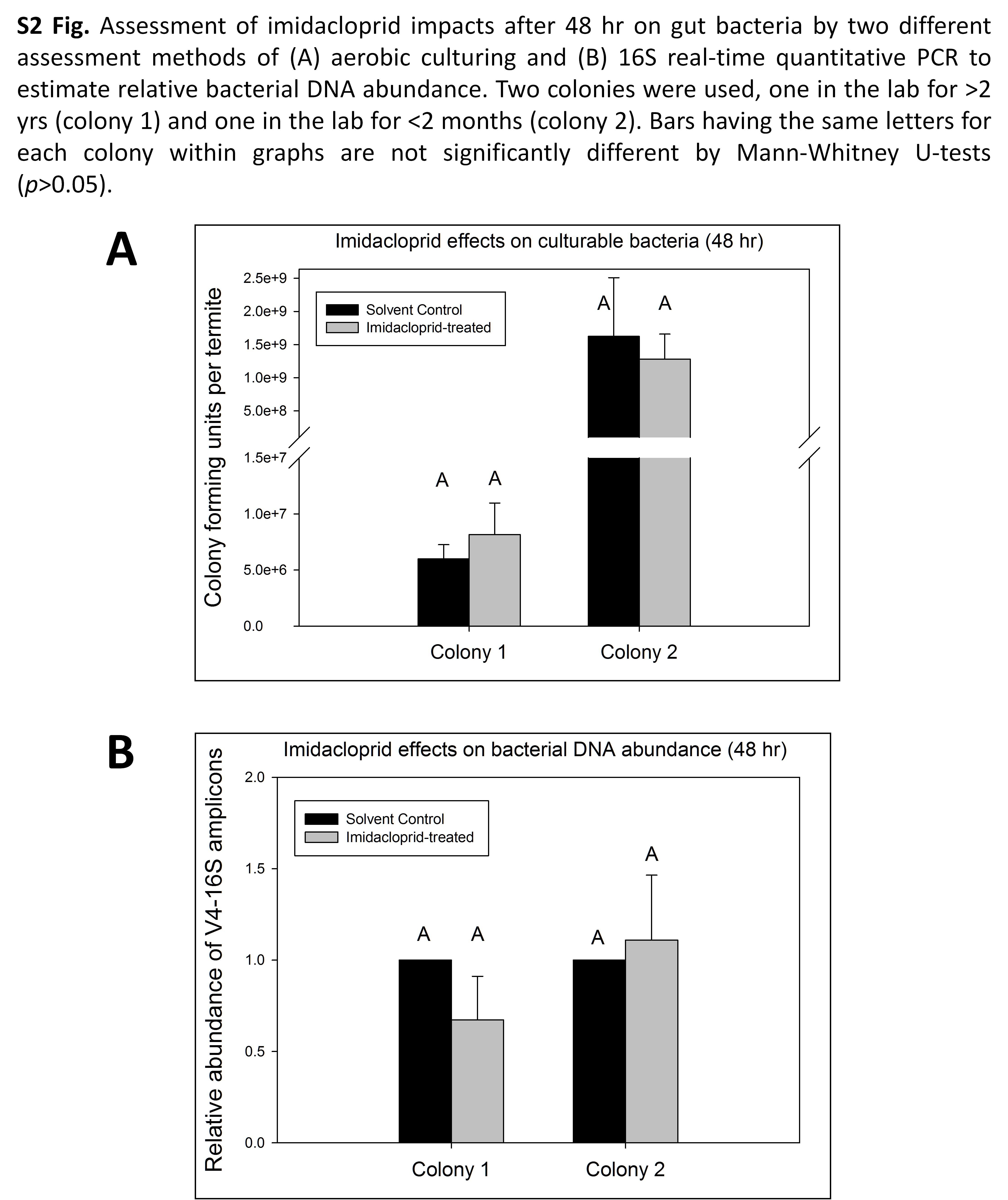

Supplement: S2 Fig — Assessment of imidacloprid impacts after 48 hr on gut bacteria by two different assessment methods of (A) aerobic culturing and (B) 16S real-time quantitative PCR to estimate relative bacterial DNA abundance. Two colonies were used, one in the lab for >2 yrs. (colony 1) and one in the lab for <2 months (colony 2). Bars having the same letters for each colony within graphs are not significantly different by Mann-Whitney U-tests (p>0.05). (JPG) [file pone.0123391.s002.jpg]

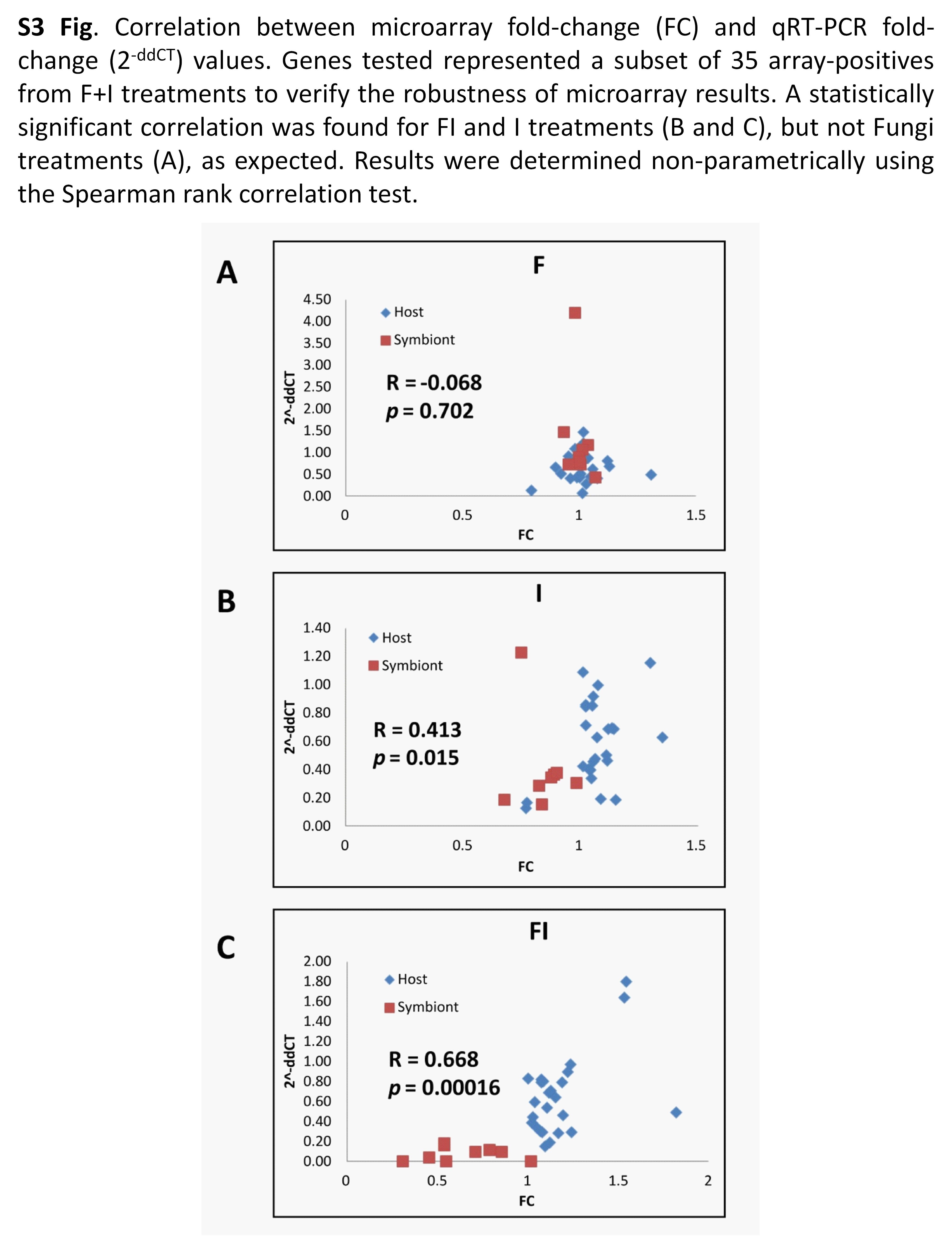

Supplement: S3 Fig — Correlation between microarray fold-change (FC) and qRT-PCR fold-change (2-ddCT) values. Genes tested represented a subset of 35 array-positives from F+I treatments to verify the robustness of microarray results. A statistically significant correlation was found for FI and I treatments (B and C), but not F treatments (A), as expected. (JPG) [file pone.0123391.s003.jpg]

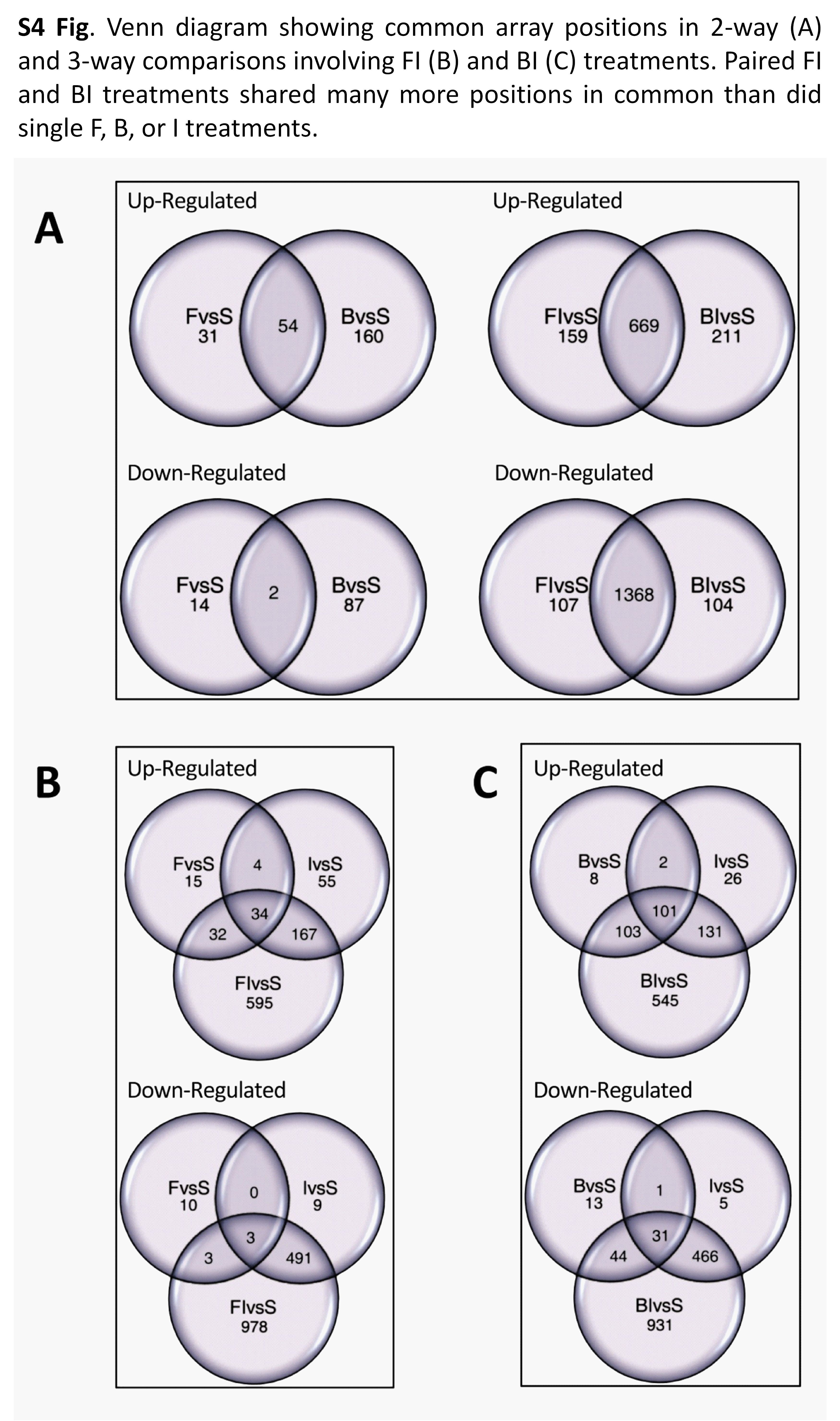

Supplement: S4 Fig — Venn diagram showing common array positions in 2-way (A) and 3-way comparisons involving FI (B) and BI (C) treatments. Paired FI and BI treatments shared many more positions in common than did single F, B, or I treatments. (JPG) [file pone.0123391.s004.jpg]

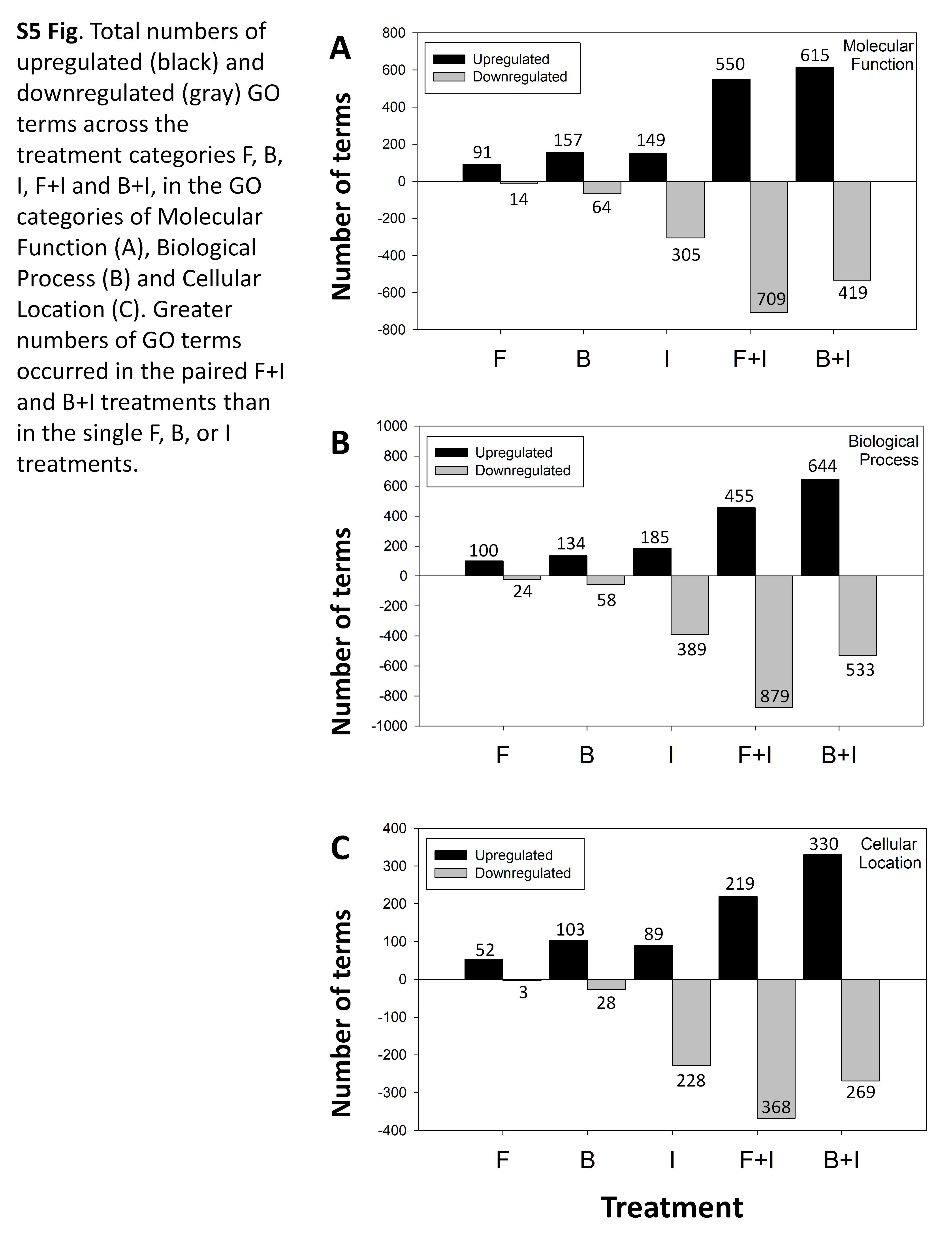

Supplement: S5 Fig — Total numbers of upregulated (black) and downregulated (gray) GO terms across the treatment categories F, B, I, F+I and B+I, in the GO categories of Molecular Function (A), Biological Process (B) and Cellular Location (C). Greater numbers of GO terms occurred in the paired F+I and B+I treatments than in the single F, B, or I treatments. (JPG) [file pone.0123391.s005.jpg]
